# Headache medication and the COVID-19 pandemic

**DOI:** 10.1186/s10194-020-01106-5

**Published:** 2020-04-25

**Authors:** Antoinette MaassenVanDenBrink, Tessa de Vries, A. H. Jan Danser

**Affiliations:** Dept. of Internal Medicine, Division of Pharmacology and Vascular Medicine, Erasmus MC University Medical Center Rotterdam, P.O. Box 2040, 3000 CA Rotterdam, The Netherlands

**Keywords:** COVID-19, Corona virus, Headache, Migraine, RAS inhibitors, Candesartan, Ibuprofen

## Abstract

The world is currently dominated by the Corona Virus Disease 2019 (COVID-19) pandemic. Besides the obvious concerns about limitation of virus spread and providing the best possible care to infected patients, a concomitant concern has now arisen in view of a putative link between the use of certain drugs, such as Renin-Angiotensin System (RAS) inhibitors and ibuprofen, and an increased risk for COVID-19 infection. We here discuss this concern in relation to headache treatment and conclude that, based on current evidence, there is no reason to abandon treatment of headache patients with RAS inhibitors or ibuprofen.

## Background

**Fig. 1:**
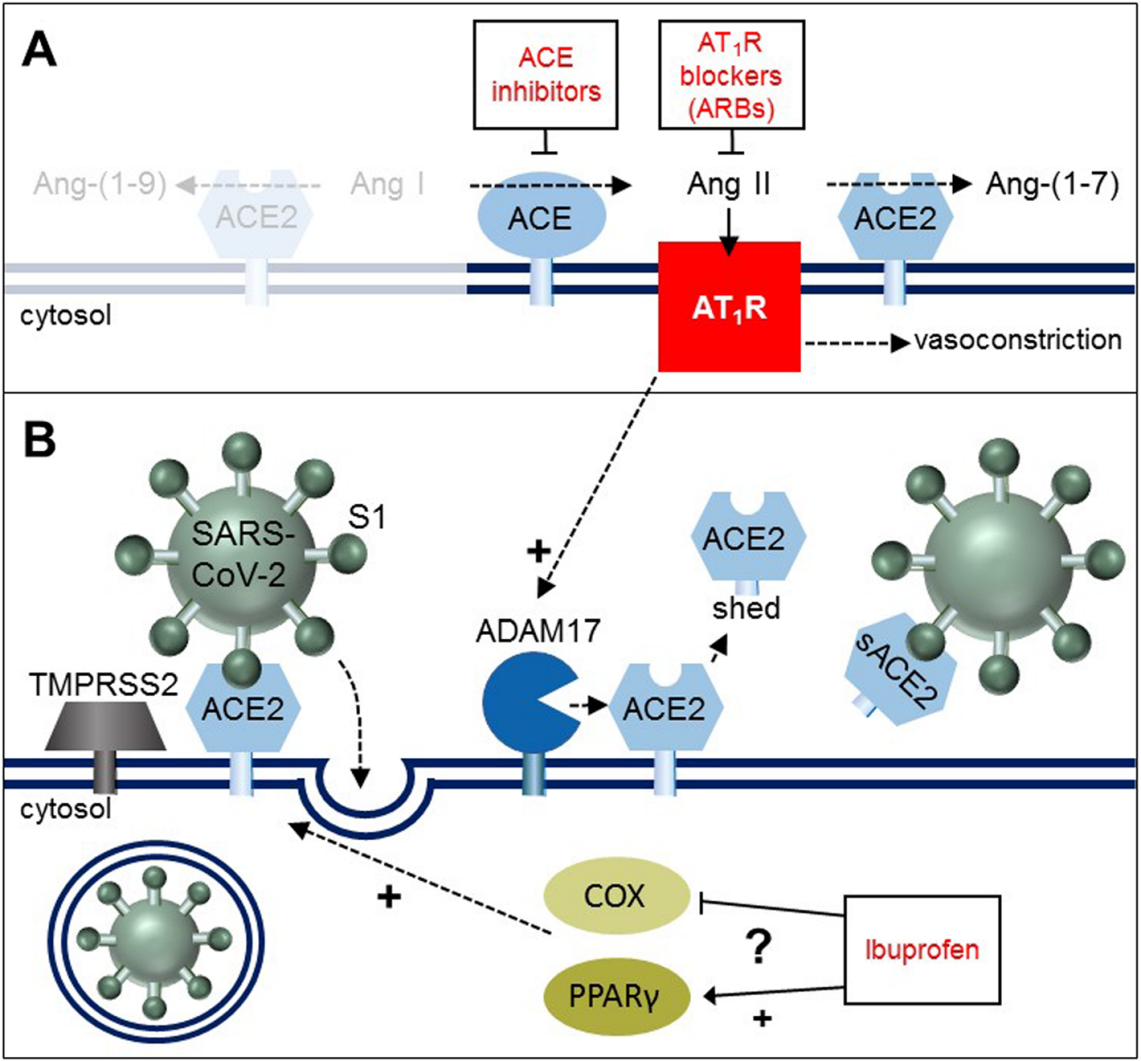
The carboxypeptidase angiotensin-converting enzyme 2 (ACE2) converts angiotensin (Ang) I into Ang-(1–9) and Ang II into Ang-(1–7) and (panel **a**), yet is not blocked by ACE inhibitors, which prevent the conversion of Ang I to Ang II. As depicted in (panel **b**), ACE2 also binds and internalizes SARS-Cov-2, after priming by the serine protease transmembrane protease, serine 2 (TMPRSS2). Shedding of membrane-bound ACE2 by a disintegrin and metalloprotease 17 (ADAM17) results in the occurrence of soluble (s) ACE2, which can no longer mediate SARS-Cov-2 entry, and which might even prevent such entry by keeping the virus in solution. Ang II, via its type 1 receptor (AT_1_R), upregulates ADAM17, and AT_1_R blockers (ARBs) would prevent this. Ibuprofen has been suggested to increase ACE2, possibly via inhibition of cyclooxygenases (COXs) and activation of Peroxisome Proliferator-Activated Receptor gamma (PPAR-γ). Redrawn after [4]

The world is currently dominated by the pandemic spread of the severe acute respiratory syndrome coronavirus-2 (SARS-CoV-2), which has already infected almost 2,000,000 people worldwide, leading to more than 120,000 deaths (for actual number, see https://who.sprinklr.com/). Whereas the priority of health authorities is to limit the spread of this virus and to provide the best possible care for patients [1], this pandemic also has consequences for the treatment of other diseases, such as headache. A statement from the European Headache Federation on how to currently treat headache patients (often from a distance) has recently been published (https://twitter.com/EMHAlliance/status/1243096347731001344). A concomitant concern has now arisen in view of a putative link between the use of certain drugs and an increased risk for COVID-19 infection [2]. This particularly applies to renin-angiotensin system (RAS) blockers and the non-steroid anti-inflammatory drug (NSAID) ibuprofen, and is based on the idea that these drugs upregulate the expression of Angiotensin-Converting Enzyme (ACE) 2, the receptor which facilitates SARS-CoV-2 entry [3]. Such entry depends on priming by the serine protease transmembrane protease, serine 2 (TMPRSS2) (Fig. 1).

RAS blockers are currently widely used as off-label drugs in the prophylactic treatment of migraine [5]. This mainly concerns the angiotensin-converting enzyme (ACE) inhibitors captopril and lisonopril and the angiotensin II type 1 receptor (AT1R) blocker (ARB) candesartan [6]. Ibuprofen is also widely used in the treatment of migraine [6], as well as in other types of headache or pain in general, because of its strong analgesic properties.

Apart from the usual considerations, such as drug-drug interactions or gastrointestinal safety the NSAIDs [7], in view of the suggested increased risk for COVID-19 infection by RAS inhibitors and ibuprofen, many clinicians now need to know what to advice their patients: continue treatment or not. The relation between RAS blockade and the COVID-19 pandemic in hypertensive patients has recently been discussed, and the advice was to continue RAS blocker treatment in such patients [4]. In this short communication, we discuss this concern in relation to headache treatment. We conclude that, based on current evidence, there is no reason to abandon treatment of headache patients with RAS inhibitors or ibuprofen.

## Renin-angiotensin system (RAS) blockers and ACE2

The renin-angiotensin system is pivotal in the regulation of blood pressure. One of its main components is ACE, which converts angiotensin I (Ang I) into angiotensin II (Ang II). Ang II exerts its hypertensive effects via AT_1_R activation. Apart from ACE, there are multiple other enzymes that metabolize angiotensin (the so-called “angiotensinases”), and one of these is the carboxypeptidase angiotensin-converting enzyme 2 (ACE2). ACE2 converts the octapeptide Ang II (=Ang-(1–8)) into Ang-(1–7), and the decapeptide Ang I (=Ang-(1–10)) into Ang-(1–9) (Fig. 1). Yet, it additionally hydrolyzes multiple other peptides beyond the angiotensins. It is important to note that ACE2 does not convert Ang I into Ang II, and that its activity is not blocked by ACE inhibitors. This is not surprising, since ACE2 and ACE are different enzymes, and ACE inhibitors have been designed specifically for ACE only. ACE2 is a membrane-bound enzyme, with very low (soluble) levels in blood [8, 9]. The occurrence of sACE2 depends on cleavage of its membrane-anchor by A Disintegrin And Metalloprotease 17 (ADAM17) (Fig. 1). Interestingly, Ang II upregulates ADAM17. Yet, given that the vast majority of ACE2 is membrane-bound, fluctuations in the percentage of sACE by pathological conditions or drug use are unlikely to have major effects on the amount of membrane-bound ACE2. Obviously, SARS-CoV-2 entry relies exclusively on membrane-bound ACE2. sACE2 cannot mediate such entry, and, if anything, might even prevent it by keeping the virus in solution.

The concern related to the use of RAS blockers in COVID-19 patients is based on the idea that these drugs upregulate ACE2. Indeed, animal studies support such ACE2 upregulation after ARB treatment [10–12]. However, this generally required high doses, while effects differed per organ and per ARB. If true, this phenomenon should also be observed for ACE inhibitors. Yet, this has hardly been studied. Most importantly, we do not know whether the increase concerned membrane-bound ACE2 in pulmonary cells (relevant in SARS patients), nor whether a rise in membrane-bound ACE2, if occurring, truly facilitates virus entry. We do know that stopping RAS blocker treatment, particular in cardiovascular patients, has major serious consequences, including an increase in mortality.

Migraine patients using RAS blockers for the prevention of migraine often additionally suffer from hypertension. Normalizing increased blood pressure protects against cardiovascular disease, while migraine, especially in women, is associated with an increased cardiovascular risk [13]. Hence, suddenly aborting preventive treatment with RAS blockers is likely to impose an increased cardiovascular risk in migraine patients, similar to that in hypertensive patients.

## Ibuprofen and ACE2

Like RAS inhibitors, ibuprofen has been suggested to increase ACE2 [14]. This conclusion is based on a study in diabetic rats exposed to one high dose of ibuprofen (40 mg/kg, corresponding with almost 3 g in a human being of 70 k). The increase in ACE2 was shown in the heart only, and no distinction was made between membrane-bound ACE2 and sACE2. Without providing evidence, the authors speculated that the ACE2 rise was due to inhibition of cyclo-oxygenase and/or activation of peroxisome proliferator-activated receptor γ [14]. Clearly, this is an exceptionally weak basis to draw a far-reaching conclusion on the use of ibuprofen in headache patients during the COVID-19 pandemic. Irrespective of this observation, we stress that paracetamol (acetaminophen) should be used as a first choice in headache treatment before starting with NSAIDs, given its better tolerability.

## Conclusion

Summarizing, there is no convincing evidence that either RAS blockers or ibuprofen facilitate or worsen SARS-CoV-2 infection in any type of patient, including headache patients. In agreement with the advice of the main cardiovascular societies (American Heart Association, https://newsroom.heart.org/news/patients-taking-ace-i-and-arbs-who-contract-covid-19-should-continue-treatment-unless-otherwise-advised-by-their-physician, European Society of Hypertension, https://www.eshonline.org/spotlights/esh-stabtement-on-covid-19/, International Society of Hypertension, https://ish-world.com/news/a/A-statement-from-the-International-Society-of-Hypertension-on-COVID-19/), the World Health Organization (https://twitter.com/WHO/status/1240409217997189128) and European Medicines Agency (https://www.ema.europa.eu/en/news/ema-gives-advice-use-non-steroidal-anti-inflammatories-covid-19), we see no rationale to panic and to alter the prescription of these drugs that have an important role in the treatment of headache.

## Data Availability

NA

## Abbreviations

ACE: Angiotensin-Converting Enzyme
ADAM17: A Disintegrin And Metalloprotease 17
Ang I: Angiotensin I
Ang II: Angiotensin II
ARB: Angiotensin II type 1 receptor blocker
AT_1_R: Angiotensin II type 1 receptor
COVID-19: Corona Virus Disease 2019
NSAID: Non-Steroid Anti-Inflammatory Drug
RAS: Renin-Angiotensin-System
SARS: Severe acute respiratory syndrome
SARS-CoV-2: Severe acute respiratory syndrome coronavirus-2
TMPRSS2: Serine protease transmembrane protease, serine 2
